# Numerical and Experimental Studies on the Improvement of Gas Chamber Structure during Gas-Assisted Extrusion

**DOI:** 10.3390/polym14235272

**Published:** 2022-12-02

**Authors:** Bin Liu, Xingyuan Huang, Xiaohui Zhang, Shaoyi Ren, Qiang Lan, Cheng Luo

**Affiliations:** Department of Mechanical Engineering, College of Advanced Manufacturing, Nanchang University, Nanchang 330031, China

**Keywords:** gas chamber structure, gas-assisted extrusion, velocity unevenness coefficient, numerical simulation

## Abstract

In the gas-assisted extrusion process, the melt inside the die is in a low-viscosity molten state, so the flow field of the gas cushion layer has a great effect on the cross-sectional shape of the micro-tube. Therefore, this study establishes the gas distribution chamber model of the gas-assisted die. Ansys Fluent software was used to simulate the gas flow field of the gas distribution chamber. The effect of the gas chamber structure on the size of the micro-tube was analyzed by the extrusion experiment. The research shows that the velocity unevenness coefficient of the gas outlet of the single gas chamber die is 11.8%, which is higher than that of the double gas chamber die. The use of a double gas chamber die can improve the stability of the gas cushion layer and the wall thickness non-uniformity of the micro-tube, which verifies the simulation results.

## 1. Introduction

Due to their small size and good plasticity, plastic micro-tubes are widely used in the medical, communication, petroleum, and automotive electronics industries [1]. With the improvement of machining technology, precision extrusion technology is widely used for the continuous machining of micro-tubes. However, the micro-scale effect has a great influence during the micro-tube extrusion process [2], which results in poor dimensional accuracy of the plastic micro-tube. Solving the problem of extrusion technology is an important means to improve the quality and dimensional qualification rate of products. At present, many scholars have studied the effects of extrusion process parameters [3,4,5,6] on the rheological characteristics [7,8,9,10,11] and extrusion expansion [12] of the melt by means of die optimization design [13,14] and numerical simulations [15,16] of the traditional extrusion process for plastic micro-tubes. However, there are some problems such as extrusion expansion, distortion and melt rupture [17,18,19] in the conventional extrusion process, which limit the processing efficiency and production cost of plastic micro-tubes.

Gas-assisted extrusion technology involving the introduction of gas between the die and the melt can change the melt in the die from adhesive flow to slip flow, which can reduce the shear stress of the melt in the die and effectively eliminate the problem of extrusion expansion and distortion [20,21,22]. Gas-assisted extrusion technology can significantly improve production efficiency, save energy consumption, and improve product size accuracy [23]. However, the influence mechanism of complex process parameters on the coupling of microtubular external dimensions is not clear, which has some limitations in practical applications. Liang [24] introduced nitrogen into the HDPE melt round bar and die through the slit to form a stable gas cushion, which effectively reduced the die pressure drop and extrusion expansion rate of the melt and improved the quality and yield. Ren [25,26,27] found that the gas-assisted technology can overcome extrusion expansion, improve the surface quality of the micro-tube, and reduce the pressure, shear stress, and first normal stress difference of the melt inside the die through experimental research and numerical analysis. Liu [28] analyzed the effect of the inner gas cushion layer pressure on the melt pressure difference and the size of the micro-tube by numerical simulation and determined the control steps to stabilize the gas cushion layer during gas-assisted extrusion through experimental studies.

In previous numerical simulations of gas-assisted extrusion processes, the gas-assisted inlet mode was simplified to an annular slit uniform inlet [29], which differs significantly from the actual single-hole inlet mode; moreover, few studies have been conducted on the effect of the gas flow field in the gas distribution chamber on the external dimensions of the micro-tube. In addition, the single-chamber die requires high gas control accuracy and complex regulation [30] to form a stable gas cushion layer. These problems limit the popularization of gas-assisted extrusion technology. In this paper, a double-chamber gas-assisted die has been designed, and the gas flow fields of single and double chambers have been analyzed by numerical simulation. Gas-assisted extrusion experiments were conducted using different chamber dies to obtain the effects of the chamber structure on the stability of the air cushion layer and microtubule deformation.

## 2. Numerical Modelling and Methods

### 2.1. Geometric and Finite Element Models

**Figure 1:**
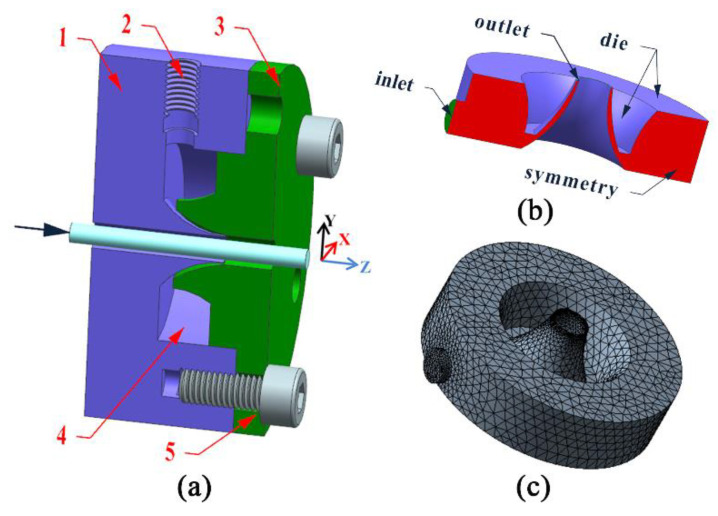
Geometric model and finite element model of the single chamber die. (**a**) Geometric model of the single-chamber gas-assisted die, 1—Connection module, 2—Gas inlet, 3—Die ring, 4—Single gas distribution chamber, 5—Connecting screw; (**b**) Geometry of the single gas distribution chamber; (**c**) Finite element mesh model of the single gas distribution chamber.

The geometry of the single gas chamber die is shown in Figure 1a. It comprises a connection module, a gas inlet, a single gas distribution chamber, and a die ring. During the gas-assisted extrusion process, the gas enters the gas distribution chamber through the gas inlet. The gas is dispersed by the blocking action of the walls in the gas distribution chamber. Finally, it flows out of the annular outlet through the gas flow channel. Then, a stable outer gas cushion layer was established between the annular melt and the inner wall of the extrusion die to assist in the melt slip extrusion. The melt flow direction is along the *Z*-axis and the gas inlet direction is along the *Y*-axis. In Figure 1b, the gas distribution chamber of the single-chamber die was established by extracting the internal flow channel. It includes a gas inlet, distribution chamber, wall, and gas outlet. The gas inlet was set as a through-hole with a diameter of 4 mm. The gas outlet was set as a ring-shaped round hole with an inner diameter of 4 mm and an outer diameter of 4.4 mm. In order to improve the calculation accuracy, the grid of the gas inlet and gas outlet was encrypted, as shown in Figure 1c.

**Figure 2:**
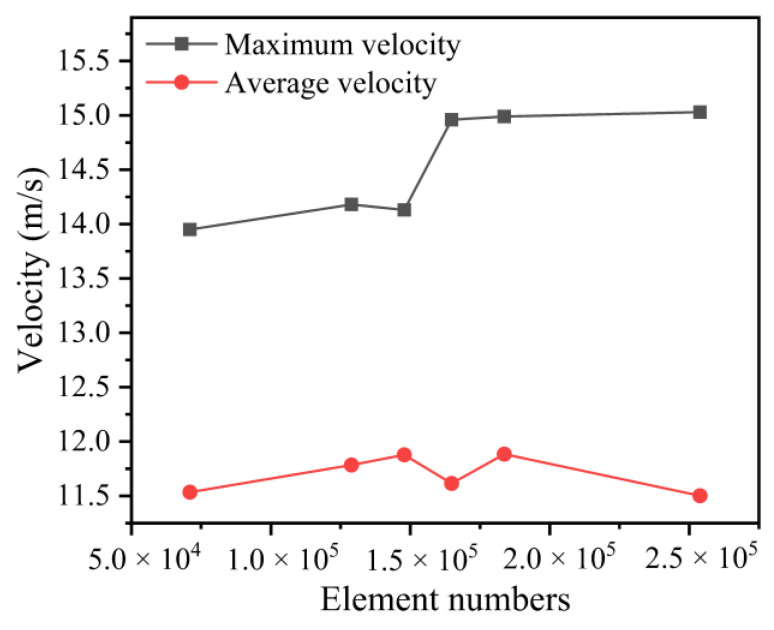
Effect of the number of grids on the simulation results.

As shown in Figure 2, when the number of meshes is less than 160,000, the maximum velocity at the gas outlet has an unstable abrupt change, and when the number of meshes is greater than 180,000, the maximum velocity and speed average value at the gas outlet do not vary much, but the calculation is time-consuming and laborious. Therefore, in order to improve the speed of numerical calculation, the number of meshes in the finite element model is taken as 180,000.

**Figure 3:**
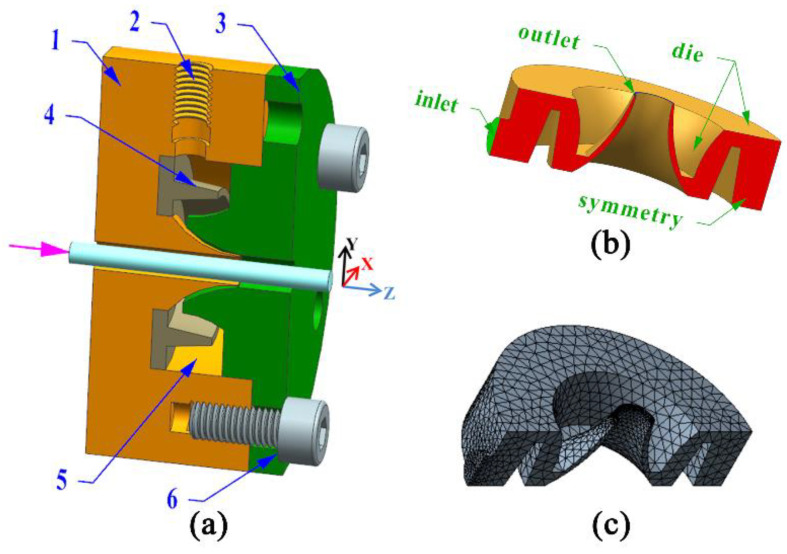
Geometric model and finite element model of the double-chamber die. (**a**) Geometric model of the double-chamber gas-assisted die, 1—Connection module, 2—Gas inlet, 3—Die ring, 4—Gas diffuser plate, 5—Double gas distribution chamber; 6—Connecting screw; (**b**) Geometry of the double gas distribution chamber; (**c**) Finite element mesh model of the double gas distribution chamber.

The geometry of the double gas chamber die is shown in Figure 3a. It includes a gas diffuser plate, a connection module, a gas inlet, a double gas distribution chamber, and a die ring. As shown in Figure 3b, the gas distribution chamber of the double-chamber die includes a gas inlet, two distribution chambers, a wall surface, and a gas outlet. The dimensions of the gas inlet, outlet, and wall of the double-chamber die are consistent with those of the single-chamber die. The gas enters the double gas distribution chamber from the gas inlet and flows out from the gas outlet after two dispersions.

### 2.2. Control Equations

The following assumptions were made during the numerical simulation process: the internal pressure and temperature of the gas distribution chamber are constant. The connection between the connection module and the die ring was well-sealed. Air was used as an auxiliary gas during the gas-assisted extrusion process. The inertial forces and gravity effects on the gas were ignored. Due to the low velocity of the gas, it was considered to be an incompressible Newtonian fluid. The effects of relative slip between the gas and the wall were ignored. The simplified gas control equations are as follows:

Continuity Equation:

(1) $$\frac{\partial u_{i}}{\partial x_{i}}+\frac{\partial u_{j}}{\partial x_{j}}+\frac{\partial u_{k}}{\partial x_{k}}=0$$

Momentum conservation equation:

(2) $$\rho \frac{\partial (u_{i}u_{j})}{\partial x_{j}}=\frac{\partial }{\partial x_{j}}\left[\mu \left(\frac{\partial u_{i}}{\partial x_{j}}+\frac{\partial u_{j}}{\partial x_{i}}\right)-\frac{2}{3}\mu \delta_{ij}\frac{\partial u_{k}}{\partial x_{k}}\right]-\frac{\partial p}{\partial x_{i}}$$

where ρ is the density, $u_{i}$, $u_{j}$, $u_{k}$ (*i* = 1, *j* = 2, *k* = 3) are the velocity vectors, p is the pressure, μ is the viscosity of the gas, $x_{i}$, $x_{j}$, $x_{k}$ are the coordinate components, and $\delta_{ij}$ (when *i* = *j*, $\delta_{ij}=1$; when *i* ≠ *j*, $\delta_{ij}=0$) is the function.

Previous studies [31,32,33,34] have verified the feasibility of using the k−ε equation to predict the gas velocity distribution in a simple mold by CFD simulations. Therefore, the k−ε equation [32,35] was used to describe the gas flow field in the gas distribution chamber as follows:

(3) $$\frac{\partial (\rho k)}{\partial t}+\frac{\partial (\rho ku_{i})}{\partial x_{i}}=\frac{\partial }{\partial x_{j}}\left[\left(\mu +\frac{\mu_{t}}{\sigma_{k}}\right)\frac{\partial k}{\partial x_{j}}\right]+G_{k}-\rho \varepsilon$$

(4) $$\frac{\partial (\rho \varepsilon )}{\partial t}+\frac{\partial (\rho \varepsilon u_{i})}{\partial x_{i}}=\frac{\partial }{\partial x_{j}}\left[\left(\mu +\frac{\mu_{t}}{\sigma_{\varepsilon }}\right)\frac{\partial k}{\partial x_{j}}\right]+C_{1\varepsilon }\frac{\varepsilon }{k}G_{k}-C_{2\varepsilon }\rho \frac{\varepsilon^{2}}{k}$$

(5) $$G_{k}=-\rho {u_{i}}^{\prime }{u_{j}}^{\prime }\frac{\partial u_{i}}{\partial x_{i}}$$

(6) $$\mu_{t}=\rho C_{\mu }\frac{k^{{}_{2}}}{\varepsilon }$$

where k is the turbulent kinetic energy, ε is the turbulence dissipation rate, $C_{1\varepsilon }$ and $C_{2\varepsilon }$ are the empirical constants of the ε equation, $G_{k}$ is the generation of turbulence kinetic energy, $\mu_{t}$ is the eddy viscosity, $\sigma_{k}$ is the empirical constant of the k equation, $\sigma_{\varepsilon }$ is the empirical constant of the ε equation, $C_{\mu }$ is the empirical constant of $\mu_{t}$, and $u_{i}$ is the component of the velocity vector. The empirical constants embedded in these equations are as follows: $\sigma_{k}=1.0$, $\sigma_{\varepsilon }=1.3$, $C_{\mu }=0.09$, $C_{1\varepsilon }=1.44$, and $C_{2\varepsilon }=1.92$.

### 2.3. Boundary Conditions

The melt flow direction is along the *Z*-axis, the gas inlet direction is along the *Y*-axis, and the gas outlet direction is along the *Z*-axis. The boundary conditions are as follows:

(1) Gas inlet boundary: the inlet boundary is set as the velocity inlet boundary. The gas inlet velocities are set to 2, 4, 6 and 8 m/s, respectively. The temperature at the inlet boundary is set to 210 °C.
(2) Wall boundary: the wall boundary is regarded as a non-slip, rigid, and static interface. The temperature is set to 210 °C.
(3) Gas outlet boundary: During the gas-assisted extrusion process, the gas is distributed around the melt in a ring shape after flowing out of the gas outlet boundary, which forms a stable gas cushion layer. Therefore, the gas outlet boundary is set as the outflow boundary.

### 2.4. Numerical Methods

In the numerical simulation, Siemens NX was used for establishing the geometry of the gas-assisted extrusion die and gas distribution chamber, ANSYS Workbench was used for fluid analysis, Geometry module was used for importing and optimizing the geometry model, the mesh module was used for loading the computational domain and meshing, and Fluent module was used for performing the gas flow field analysis. The pressure-based solver was used for the boundary solution method, the absolute velocity was selected for the velocity equation, the steady-state flow was selected for the time solution type, and the Simple algorithm was used to solve the discrete equation.

### 2.5. Evaluation Indicators

In order to analyze quantitatively the gas flow field at the outlet boundary of the gas distribution chamber and compare the effect of the gas distribution chamber structure on the micro-tube, the velocity unevenness coefficient [36] at the outlet boundary of the gas distribution chamber is used as an evaluation indicator. The formula is as follows:

(7) $$M_{v}=\frac{\sqrt{\frac{1}{n-1}\sum {\left(v-\bar{v}\right)}^{2}}}{\bar{v}}\times 100\%$$

where $M_{v}$ is the velocity unevenness coefficient at the outlet boundary of the gas distribution chamber, v is the normal velocity at each calibration point at the gas outlet boundary, $\bar{v}$ is the average velocity at each point, and n is the number of calibration points.

**Figure 4:**
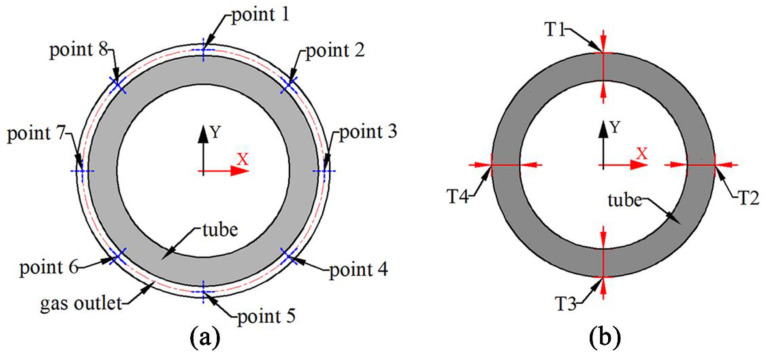
Schematic diagram of the calibration point at the gas outlet boundary. (**a**) Location plot of the calibration points at the gas outlet boundary; (**b**) Location plot of the micro-tube thickness measurement.

In order to calculate the unevenness coefficient more accurately, 8 calibration points were uniformly selected at the gas outlet boundary as the value position of the normal velocity. As shown in Figure 4, the center of the gas outlet boundary is the origin of the X-Y coordinate. In these cases, the X-Y coordinate values of each point are shown in Table 1.

## 3. Simulation Results and Discussion

### 3.1. Results of Velocity Fields

**Figure 5:**
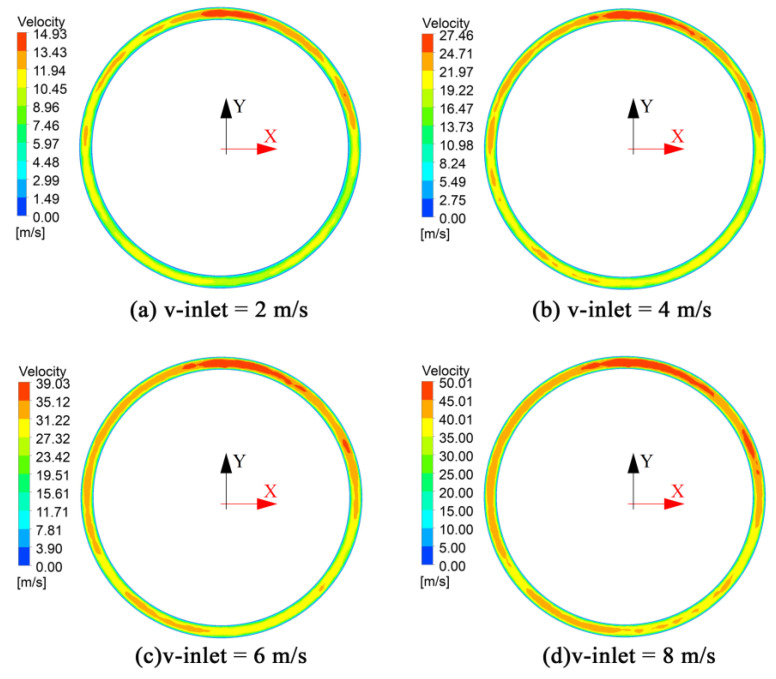
Distribution of gas flow velocity at the gas outlet boundary of the single gas chamber die. (**a**) Gas inlet flow velocity of 2 m/s; (**b**) Gas inlet flow velocity of 4 m/s; (**c**) Gas inlet flow velocity of 6 m/s; (**d**) Gas inlet flow velocity of 8 m/s.

**Figure 6:**
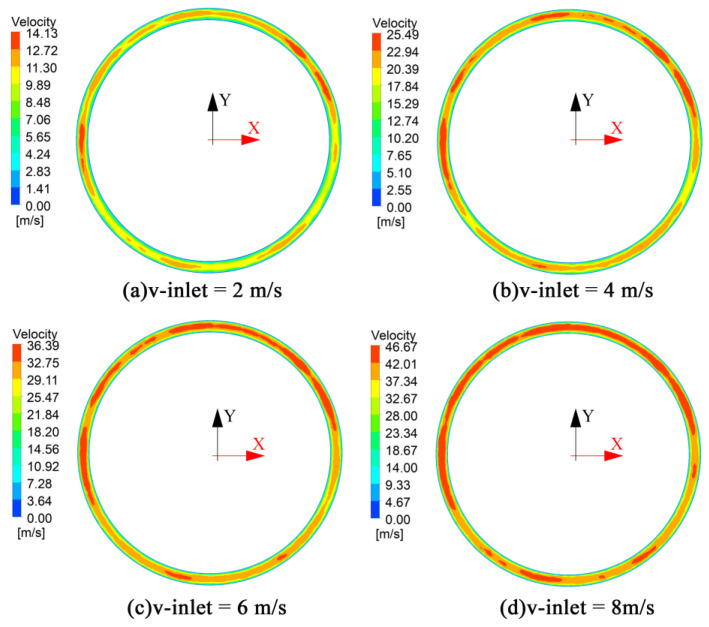
Distribution of gas flow velocity at the gas outlet boundary of the double gas chamber die. (**a**) Gas inlet flow velocity of 2 m/s; (**b**) Gas inlet flow velocity of 4 m/s; (**c**) Gas inlet flow velocity of 6 m/s; (**d**) Gas inlet flow velocity of 8 m/s.

The distribution of gas flow velocities at the outlet of the dies with different gas chambers is shown in Figure 5 and Figure 6.

It can be seen from Figure 5 that the distribution of the gas normal velocity of the single chamber die at the gas outlet boundary is uneven. Therefore, the flow rate distribution at the gas outlet boundary is uneven. There is a maximum velocity position in the positive direction of the *Y*-axis. This is because after the gas enters the gas distribution chamber from the inlet along the positive direction of the *Y*-axis, most of the gas flows out of the bottom of the gas distribution chamber along the shortest path of the gas flow channel, resulting in the maximum velocity at this position. Less gas is obstructed by the inner wall of the die to complete the dispersion. As the gas inlet velocity increases from 2 m/s to 8 m/s, the maximum gas outlet velocity increases linearly, but at the same position.

From Figure 6, it can be seen that the gas normal velocity distribution of the double-chamber die at the gas outlet boundary is relatively uniform. The maximum gas outlet velocity increases with the increase in the gas inlet velocity, and there is no obvious distribution pattern at the position of the maximum gas outlet velocity in the double-chamber die. This is because the diffuser plate has a certain dispersion effect on the gas flow. When the gas flows into the first gas chamber, its movement direction changes from *Y*-axis to the annular flow due to the blocking effect of the diffuser plate, which completes the first gas flow distribution process. Moreover, the gas is secondly dispersed by the blocking effect of the wall of the second gas chamber and then flows out from the gas outlet. Therefore, the gas flow field of the double-chamber die is more evenly distributed than that of the single-chamber die. The maximum gas outlet velocity of the double-chamber die is less than that of the single-chamber die.

### 3.2. Results of Velocity Unevenness Coefficient

**Figure 7:**
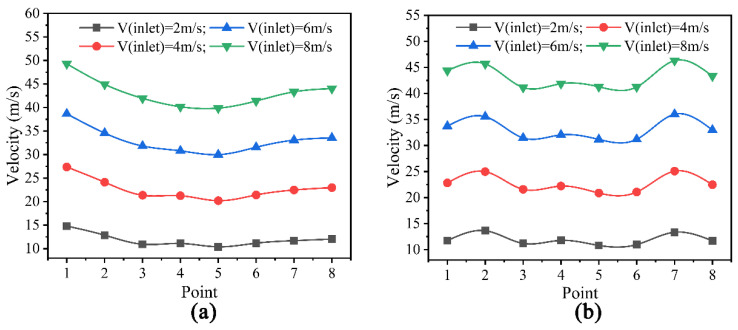
Distribution of gas outlet velocities for each calibration point. (**a**) Gas outlet velocity for a single gas chamber die; (**b**) Gas outlet velocity for a double gas chamber die.

**Figure 8:**
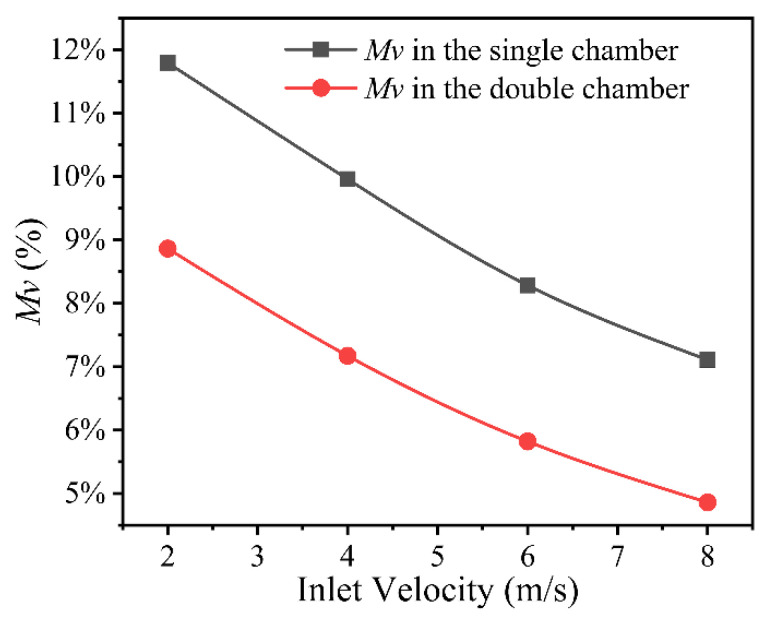
Gas outlet velocity unevenness coefficient.

The gas outlet velocities at each calibration point for different gas chamber dies are shown in Figure 7. The gas outlet velocity unevenness coefficient is shown in Figure 8.

As shown in Figure 7a, point 1 is the position of the maximum outlet velocity of the single-chamber die head. As the gas inlet velocity increases from 2 to 8 m/s, the difference between the maximum and minimum outlet velocities increases from 4.44 to 9.5 m/s. The gap between the outlet velocities at each point increases with the increase in the gas inlet velocity, but the velocity distribution trend at each point remains unchanged. It can be seen From Figure 7b that the difference between the maximum and minimum outlet velocities increases from 2.8 to 5 m/s when the gas inlet velocity of the double-chamber die increases from 2 to 8 m/s. The results show that the difference between outlet velocities at each point of the double-chamber die is smaller than that of the single-chamber die as the gas inlet velocity increases.

From Figure 8, it can be seen that the outlet velocity unevenness coefficient of the double gas chamber die is smaller than that of the single gas chamber die, which indicates that the uniform gas flow distribution effect of the double gas chamber die is better than that of the single gas chamber die. As the gas inlet velocity increases from 2 to 8 m/s, the gas outlet velocity unevenness coefficient decreases for both structured chamber dies. There are local differences in the gas outlet flow rates of both structured chamber dies.

## 4. Experimental Results and Discussion

**Figure 9:**
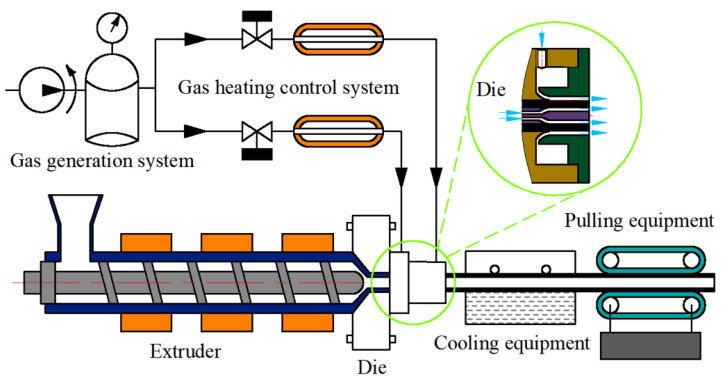
Gas-assisted extrusion experimental equipment.

**Figure 10:**
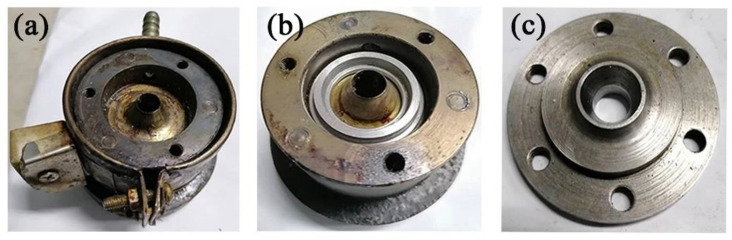
Physical diagram of the extrusion die. (**a**) Connection module for single gas chamber die; (**b**) Connection module for double gas chamber die; (**c**) Die ring.

**Figure 11:**
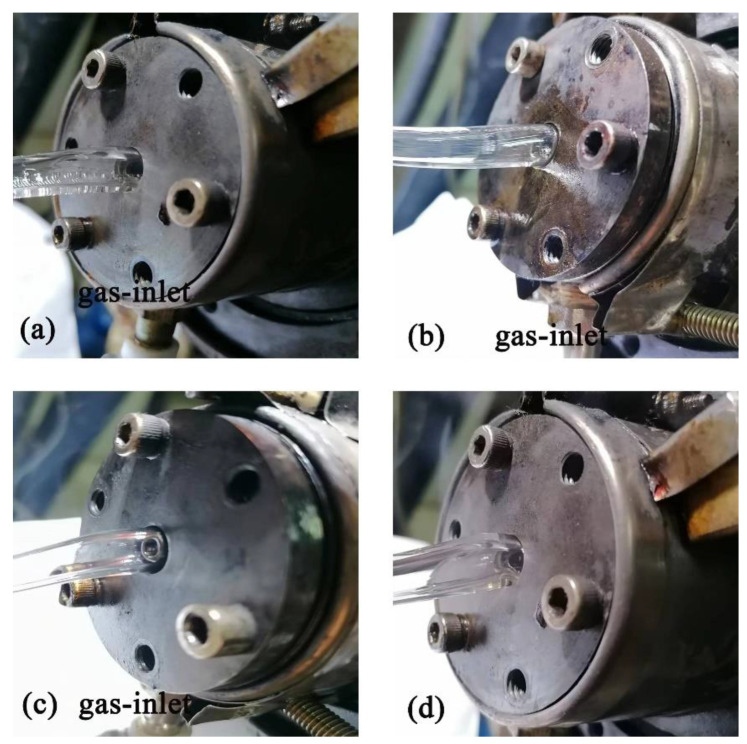
Results of extrusion experiment. (**a**) Gas inlet volume flow rate of 3 × 10^−5^ m^3^/s for the single gas chamber die; (**b**) Gas inlet volume flow rate of 3 × 10^−5^ m^3^/s for the double gas chamber die; (**c**) Gas inlet volume flow rate of 6 × 10^−5^ m^3^/s for the double gas chamber die; (**d**) Non-gas-assisted extrusion.

**Figure 12:**
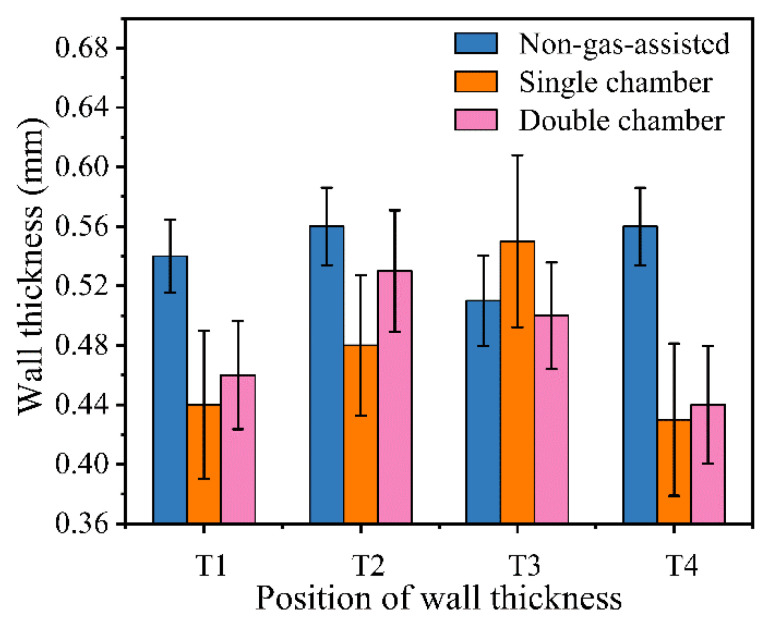
Wall thickness distribution of plastic micro-tubes for gas inlet volume flow rate of 6 × 10^−5^ m^3^/s.

The gas-assisted extrusion experimental equipment [37] is shown in Figure 9. It includes a gas generation system, a gas heating control system, an extruder, cooling equipment, and pulling equipment. The actual extrusion die is shown in Figure 10. The extrusion experimental material was polypropylene (PPH-T03, produced by Sinopec Co., Ltd., Beijing, with a melting point of 164 °C and a density of 900 kg/m^3^). The purpose of the experiment was to verify the effect of the gas chamber structure on the microtubule size. As a result, the melt inlet flow rate, melt temperature, extruder speed, and pulling speed were set to be the same, as shown in Table 2. The results of the extrusion experiment are shown in Figure 11. The wall thickness distribution of the plastic micro-tube is shown in Figure 12.

It can be seen from Figure 11a that during the extrusion process of the single gas chamber die, when the gas inlet volume flow rate is 3 × 10^−5^ m^3^/s, there are obvious ripples on the melt surface near the gas inlet, while the melt surface at other positions adheres to the wall of the die. This indicates that no stable gas cushion layer is formed around the melt, the auxiliary gas is concentrated to flow out near the gas inlet, and the melt at this position is disturbed by obvious airflow, which squeezes the melt to a low wind position speed and makes the surrounding melt adhere to the surface of the die. Confirming the numerical simulation results in Figure 5, the maximum position of the gas outlet velocity is near the position of the gas inlet.

As can be seen in Figure 11b, there is a clear gap between the melt and the wall of the double gas chamber die at an inlet volume flow rate of 3 × 10^−5^ m^3^/s. This indicates that a stable gas cushion layer is formed around the melt. The gas flow parameters for the formation of a stable gas cushion layer in the double gas chamber die are larger than in the single gas chamber die. It is easier to form a stable gas cushion layer using a double gas chamber die.

It can be seen from Figure 11b,c that the gap between the melt surface and the die wall increases with increasing gas inlet flow, and the outer diameter of the micro-tube decreases with the increase in the gas flow rate. This is because the dragging effect on the melt increases with increasing gas flow. Confirming the numerical simulation results in Figure 6, the gas outlet velocity increases with the increase in the gas inlet velocity. As can be seen in Figure 11d, there is an obvious extrusion expansion in the wall thickness of the plastic micro-tube during the non-gas-assisted extrusion process.

From Figure 12, it can be seen that the difference in micro-tube wall thickness is greater for gas-assisted extrusion than for non-gas-assisted extrusion. This is due to the dragging effect of the unevenly flowing auxiliary gas in the die on the melt, which leads to a local differential distribution of the micro-tube wall thickness. The difference in micro-tube wall thickness for the double gas chamber die is smaller than that for the single gas chamber die, which is consistent with the effect of the gas chamber structure on the gas flow field obtained from numerical simulations. The experimental results in this paper are consistent with the findings of Ren [38] that a phenomenon of thinning of the melt by the auxiliary gas exists during the process of gas-assisted extrusion of a plastic micro-tube. The axial outlet velocity of the melt increases with an increasing gas inlet flow rate. The outer diameter and wall thickness of the micro-tube decrease with the increase in the gas inlet flow rate.

## 5. Conclusions

1. The numerical simulation of the gas flow field in the gas distribution chamber shows that the gas chamber structure has a great influence on the unevenness coefficient of the gas outlet velocity. The outlet gas flow field of the double gas chamber die is more uniform than that of the single gas chamber die. The outlet velocity unevenness coefficient can be reduced with the increase in the gas inlet flow rate. The outlet velocity unevenness coefficient is 3% higher for the single gas chamber die than for the double gas chamber die. When the gas inlet velocity increases from 2 to 8 m/s, the outlet velocity unevenness coefficient decreases by 4%.
2. In the gas-assisted extrusion process of a plastic micro-tube, the flow field of the auxiliary gas has a great impact on the wall thickness uniformity of the plastic micro-tube. Using a double gas chamber die, the formation of a gas cushion layer is easier and more stable, which can improve the wall thickness uniformity of the plastic micro-tube.

## Figures and Tables

**Table 1:** X-Y coordinate values for each calibration point.

Calibration Point	Point 1	Point 2	Point 3	Point 4	Point 5	Point 6	Point 7	Point 8
X (mm)	0	1.4849	2.1	1.4849	0	−1.4849	−2.1	−1.4849
Y (mm)	2.1	1.4849	0	−1.4849	−2.1	−1.4849	0	1.4849

**Table 2:** Experimental conditions.

Experiment Conditions	Gas-Assisted Extrusion	Non-Gas-Assisted Extrusion
Gas inlet volume flow rate (m^3^/s)	3 × 10^−5^, 6 × 10^−5^	/
Temperature of the die (°C)	215	215
Extruder speed (r/min)	5	5
Temperature of the gas (°C)	210	/
Pulling speed (r/min)	4	4
Temperature of the melt (°C)	215	215

## Data Availability

Not applicable.

## References

[1] Cho S., Lee E., Jo S., Kim G.M., Kim W. (2020). Extrusion Characteristics of Thin Walled Tubes for Catheters Using Thermoplastic Elastomer. Polymers.

[2] Zhang H.G., Lamnawar K., Maazouz A., Maia J.M. (2016). A nonlinear shear and elongation rheological study of interfacial failure in compatible bilayer systems. J. Rheol..

[3] Liu W., Kim W., Park J.M. (2018). Numerical Study on the Optimization of Polymer Extrusion Process for a Single-Lumen Micro Catheter. Trans. Korean Soc. Mech. Eng. A.

[4] Tseng H.H. (1991). Software Design and Experimental Verification of Polymer Flow through a Pipe Extrusion Die. Ph.D. Thesis.

[5] Sahmel O., Siewert S., Arbeiter D., Kreiner C.F., Guthoff R., Schmitz K.P., Grabow N. (2019). Extrusion as a manufacturing process for polymer micro-tubes for various bio-medical applications. Curr. Dir. Biomed. Eng..

[6] Deng X.Z., Xiao B., Tang G., Yan X.X., Yu S.F. (2018). Effect of shaping length on lolymer precision extrusion for Micro Tube. Plastics.

[7] Luo X.L., Mitsoulis E. (1989). Memory phenomena in extrudate swell simulations for annular dies. J. Rheol..

[8] Kim J.H., Lyu M.Y. (2014). Predictions of flow behaviors and entrance pressure drop characteristics of a rubber compound in a capillary die using various rheological models. Polym. Eng. Sci..

[9] Chien R.D., Jong W.R., Chen S.C. (2005). Study on rheological behavior of polymer melt flowing through micro-channels considering the wall-slip effect. J. Micromech. Microeng..

[10] Wang M.J., Tian H.Q., Zhao D.Y. (2012). Micro-scale shear viscosity testing approach and viscosity model of polymer melts. Chin. J. Mech. Eng..

[11] Fernandes C., Fakhari A., Tukovic Z. (2021). Non-Isothermal Free-Surface Viscous Flow of Polymer Melts in Pipe Extrusion Using an Open-Source Interface Tracking Finite Volume Method. Polymers.

[12] Sombatsompop N., O-Charoen N. (2003). Extrudate swell behavior of PS and LLDPE melts in a dual die with mixed circular/slit flow channels in an extrusion rheometer. Polym. Adv. Technol..

[13] Tang D., Fang W.L., Fan X.H., Li D.Y., Peng Y.H. (2014). Effect of die design in microchannel tube extrusion. Procedia Eng..

[14] Jin G.B., Zhao D.Y., Wang M.J., Jin Y.F., Tian H.Q., Zhang J. (2015). Study on design and experiments of extrusion die for polypropylene single-lumen micro tubes. Microsyst. Technol..

[15] Nithi-Uthai N., Manas-Zloczower I. (2003). Numerical Simulation of Sharkskin Phenomena in Polymer Melts. Appl. Rheol..

[16] Zhou H.B., Fan H.H., Zhai Y.H., Peng Q. (2014). A new utility calculation model for axial flow of non-Newtonian fluid in concentric annuli. Can. J. Chem. Eng..

[17] Mu Y., Zhao G.Q., Wu X.G., Zhai J.Q. (2013). Finite-Element Simulation of Polymer Flow and Extrudate Swell Through Hollow Profile Extrusion Die with the Multimode Differential Viscoelastic Model. Adv. Polym. Technol..

[18] Baldi F., Briatico-Vangosa F., Franceschini A. (2014). Experimental study of the melt fracture behavior of filled high-density polyethylene melts. Polym. Eng. Sci..

[19] Xu X.M., Zhao G.Q., Qin S.X., Wang W.W. (2011). Numerical Simulation of Viscoelastic Extrudate Swell Through Elliptical Ring Die. Chin. J. Chem. Eng..

[20] Luo C., Huang X.Y., Liu T.K., Liu H.S. (2020). Research on Inner Gas Inflation Improvements in Double-layer Gas-assisted Extrusion of Micro-tubes. Polymers.

[21] Huang C.Y., Liu H.S., Huang X.Y., Wan Q.F., Ren Z. (2015). Numerical Simulation of Gas-assisted Extrusion Process of Microtube. China Plast. Ind..

[22] Deng X.Z., Xiao B., Ren Z. (2022). Research progress of polymer gas—Assisted extrusion. Polym. Mater. Sci. Eng..

[23] Yin H.N., Huang X.Y., Liu T.K., Song M.J. (2021). Effects of gas-assisted extrusion on slip in the cable coating process. J. Polym. Eng..

[24] Liang R.F., Mackley M.R. (2001). The gas-assisted extrusion of molten polyethylene. J. Rheol..

[25] Ren Z., Huang X.Y., Liu H.S., Deng X.Z., He J.T. (2015). Numerical and experimental studies for gas assisted extrusion forming of molten polypropylene. J. Appl. Polym. Sci..

[26] Ren Z., Huang X.Y., Liu H.S., Deng X.Z., He J.T. (2015). Non-isothermal viscoelastic numerical analysis of compressible gas-assisted polymer extrusion molding. CIESC J..

[27] Ren Z., Huang X.Y., Xiong Z.H. (2020). Experimental and numerical studies for the gas-assisted extrusion forming of polypropylene micro-tube. Int. J. Mater. Form..

[28] Liu T.K., Huang X.Y., Ren Z., Luo C., Tan J.M. (2020). Analysis of Superimposed Influence of Double Layer Gas Flow on Gas-Assisted Extrusion of Plastic Micro-Tube. Int. Polym. Process..

[29] Yin H.N., Huang X.Y., Liu H.S., Liu T.K., Luo C., Wang D.Y. (2021). Viscoelastic numerical simulation of gas-assisted extrusion of cable cladding. Polym. Mater. Sci. Eng..

[30] Liu T.K., Huang X.Y., Luo C., Wang D.Y. (2020). The Formation Mechanism of the Double Gas Layer in Gas-Assisted Extrusion and Its Influence on Plastic Micro-Tube Formation. Polymers.

[31] Xie S., Jiang G.J., Wu X.Y., Wang Y.P., Fang H.S., Shentu B.Q. (2021). Air Recirculation and Its Effect on Microfiber Spinning in Blunt-Die Melt Blowing. Fiber. Polym..

[32] Hassan M.A., Anantharamaiah N., Khan S.A., Pourdeyhimi B. (2016). Computational Fluid Dynamics Simulations and Experiments of Meltblown Fibrous Media: New Die Designs to Enhance Fiber Attenuation and Filtration Quality. Ind. Eng. Chem. Res..

[33] Shang S.S., Yu C.W., Li M.L. (2017). Numerical simulation of swirling airflow dynamics in vortex spinning. Text. Res. J..

[34] Xie S., Jiang G.J., Ye B.L., Shentu B.Q. (2020). Particle Image Velocimetry (PIV) Investigation of the Turbulent Airflow in Slot-Die Melt Blowing. Polymers.

[35] Krutka H.M., Shambaugh R.L., Papavassiliou D.V. (2003). Effects of Die Geometry on the Flow Field of the Melt-Blowing Process. Ind. Eng. Chem. Res..

[36] Li H.L., Wang C., Hu J., Yu H.M., Li Q.D., Zhang X.Y. (2018). Simulation and Optimum Design on Airflow Distribution Chamber of Pneumatic Forming Machine for Rice Seeding-growing Tray. Trans. Chin. Soc. Agric. Mach..

[37] Liu B., Huang X.Y., Ren S.Y., Luo C. (2022). Effect of Pressure Difference between Inner and Outer Gas Layer on Micro-Tube Deformation during Gas-Assisted Extrusion. Polymers.

[38] Ren Z., Huang X.Y. (2019). Effect of gas flow rate on the double gas-assisted extrusion forming of plastic pipes. IOP Conf. Ser. Earth Environ. Sci..

